# Sensitive Skin in the Genital Area

**DOI:** 10.3389/fmed.2019.00096

**Published:** 2019-05-15

**Authors:** Miranda A. Farage

**Affiliations:** The Procter & Gamble Company, Mason Business Center, Mason, OH, United States

**Keywords:** self-perceived sensitive skin, genital skin, gender differences, age differences, urogenital skin

## Abstract

When evaluating sensitive skin, it is common to focus on the face. However, it is becoming increasingly clear that individuals can have different perceptions about the degree of skin sensitivity at distinct anatomic sites. Structural variations in the skin of different body sites can contribute to differences in barrier function, which may contribute to differences in skin sensitivity. Potential triggering factors for skin sensitivity would be expected to vary by body site. For example, the face is exposed to all ambient environmental conditions in the course of daily life, and to several products (e.g., cosmetics for women) and practices (e.g., shaving for men) that may lead to adverse sensations associated with sensitive skin. In contrast, the skin of the genital area is protected from ambient environmental conditions. However, the genital area can be exposed to conditions of higher temperature, different habits and practices and moisture due to the semi-occlusive environment. For some individuals, additional challenges such as incontinence can provide triggering factors for skin sensitivity that effect only the genital area. This article reviews data on the perception of sensitive skin of the genital area, differences based on gender, age, racial differences, and the effects of incontinence on skin sensitivity. The effects of menopause are also considered with regards to sensitive skin perceptions and to emerging differences in biomolecular and physical measures of the urogenital skin.

## Introduction to “Sensitive Skin”

Individuals with sensitive skin report a variety of unpleasant sensory reactions in response to common external factors and intrinsic stressors (1, 2). Often, the sensory effects that are the hallmark of sensitive skin (such as prickling, burning, tingling, or pain) are not accompanied by erythema or other objective signs of irritation or immunological responses (1). In fact, little correlation exists between individuals' perceptions of the sensitivity of their skin and demonstrable signs of skin reactivity to irritants (3). A consensus definition of this condition was published in 2017 as follows.

“A syndrome defined by the occurrence of unpleasant sensations (stinging, burning, pain, pruritus, and tingling sensations) in response to stimuli that normally should not provoke such sensations. These unpleasant sensations cannot be explained by lesions attributable to any skin disease. The skin can appear normal or be accompanied by erythema. Sensitive skin can affect all body locations, especially the face” (4).

The pathogenesis of sensitive skin is unknown, but believed to be the product of multiple etiologies, including; deficiencies in barrier function, neurosensory dysfunction, compound-specific irritancy, and cultural influences (5, 6).

A sizeable proportion of people in the general population in many geographies claim sensitive skin (6, 7). For example, in Europe some degree of skin sensitivity was claimed by 50–90% of responders in several studies in France, (8–10) 75% of responders in Germany, (11) over 50% in Italy, (12) and 64% in Greece (13). In the UK, 38% of the men and 51% of the women claimed to have sensitive skin (14). In the US, the prevalence of self-declared sensitive skin has been reported at 44–83% (15–19). In Japan, “very” or “rather” sensitive skin is claimed by 53% of men and 56% of women (20). Sensitive skin was claimed by 57% of the subjects in Korea (21).

In other geographies, the proportion of the population who perceive they have sensitive skin is lower than in Europe and the United States. In a study conducted in Mexico using 246 subjects self-diagnosed sensitive skin was found in 36% subjects, with a higher prevalence of sensitive skin among subjects with lighter skin phototypes (Type II and III) compared to darker ones (type IV and V) (22). Two survey studies have been reported from China. In a study of 9,154 individuals the prevalence of self-proclaimed sensitive or very sensitive skin has been reported as 9% among men and 16% among women (23). In a study among 408 women in China, 2% claimed they had very sensitive skin, 5% claimed they had moderately sensitive skin, and 16% claimed they had slightly sensitive skin (24).

The explanation for differences in prevalence between countries regarding the perception of sensitive skin may be related to some of the underlying physiological causes and environmental triggers for sensitive skin, such as prevailing weather conditions, and fairer vs. darker skin type. Also, it is likely cultural influences account for some of this difference. In the study conducted in urban areas in China, Xu et al. hypothesized that some of the participants, especially older individuals, were not familiar with the concept of “sensitive skin” and, therefore, the condition may have been under-reported (23). This hypothesis was supported by the observations that some individuals who did not claim sensitive skin responded that they experienced adverse sensory effects after using cosmetic products. Further, the reported prevalence was inversely proportional to the age group of the responders.

The expectations of the general public may also play a role. Manufacturers of consumer products have increasingly marketed products targeted for sensitive skin. Consequently, the public has likely become more aware of this condition. This may partially explain why the proportion of the population that claims sensitive skin appears to be increasing (24). Results of a study conducted in eight European countries are consistent with a cultural component (25, 26). In Portugal, Italy, and Spain, 80–90% of the subjects in the survey population reported at least some skin sensitivity, while in Germany, Belgium, and Switzerland the proportion was just a little more than half. Since the European population is considered to be highly mobile and crossbred, the authors attributed this unexpected finding to substantially more fashion and beauty-related advertising in specific European countries (26).

## Unique Features of Genital Skin

When evaluating sensitive skin, it is common to focus on the face. However, it is becoming increasingly clear that individuals can have different perceptions about the degree of skin sensitivity at distinct anatomic sites (1, 7, 16). Structural variations in the skin of different body sites can contribute to differences in barrier function, which may contribute to differences in skin sensitivity. Also, potential triggering factors for skin sensitivity would be expected to vary by body site. For example, the face is exposed to all ambient environmental conditions in the course of daily life, and to several products (e.g., cosmetics for women) and practices (e.g., shaving for men) that may lead to adverse sensations associated with sensitive skin. In contrast, the skin of the genital area is more protected from ambient environmental conditions, but this anatomic site is almost constantly semi-occluded throughout the day.

**Figure 1:**
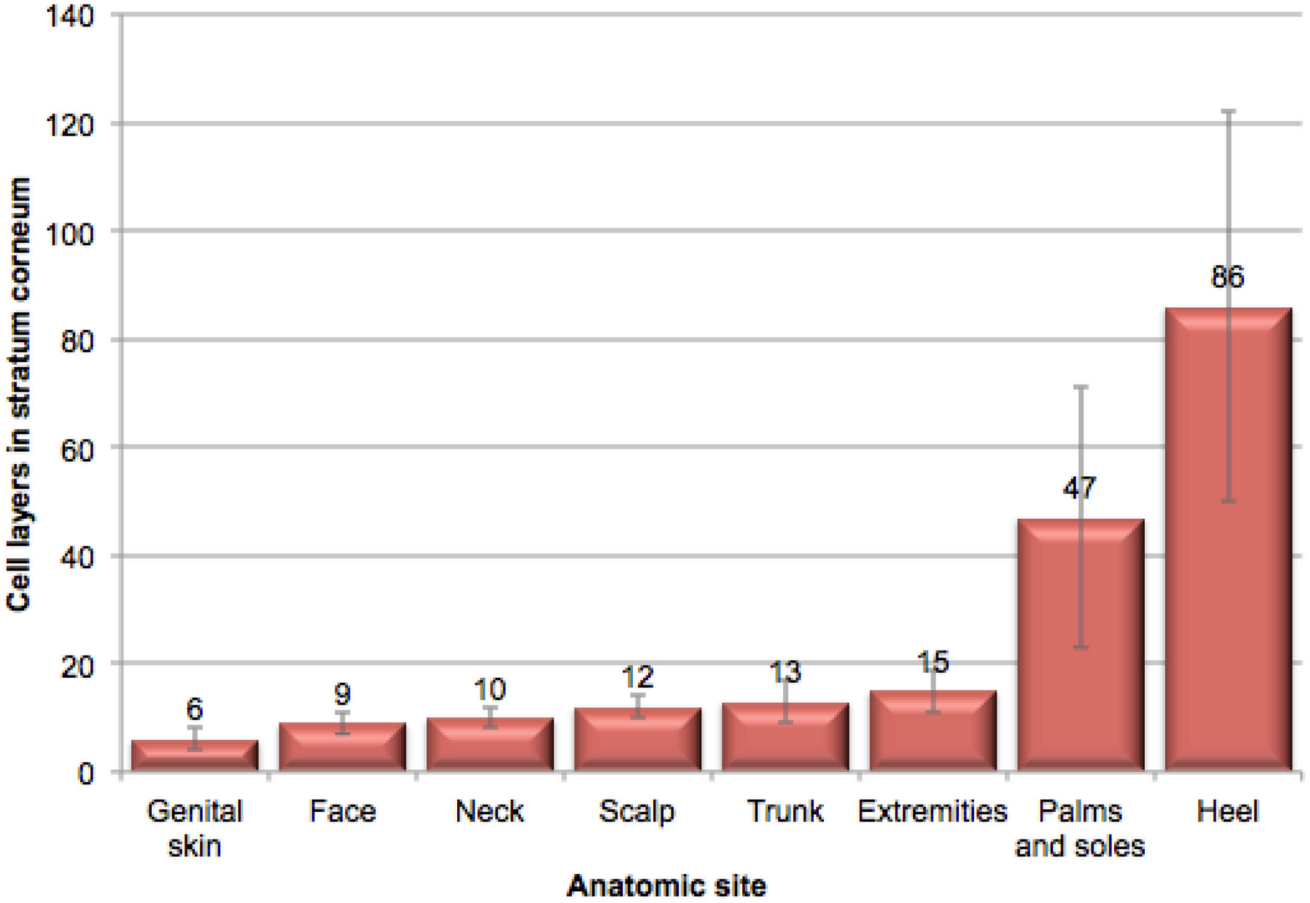
Illustration of stratum corneum thickness at various anatomic sites. This illustration of the relative thickness of stratum corneum at various anatomic sites was adapted from data in Ya-Xian et al. (28).

The structure of the stratum corneum (SC) varies depending on the anatomic site. The skin of the genital area is the only anatomic site other than the face where mucous membranes are exposed to the outside (27). It is highly innervated, vascularized, and has numerous active skin appendages (27). Ya-Xian et al. investigated the thickness of the SC from various body sites (results are illustrated in Figure 1) (28). These investigators found that the smallest number of cell layers was found in genital skin (6 ± 2), followed by skin of the face (9 ± 2), neck (10 ± 2), scalp (12 ± 2), trunk (13 ± 4), and extremities (15 ± 4). The palms and soles showed 47 (±24) cell layers, and the heel showed 86 (±36). These same investigators demonstrated that transepidermal water loss (TEWL), decreased and the thickness of the SC increased (28). Tagami reported that TEWL on facial skin was measured at 10 g/m^2^/h compared to 7 g/m^2^/h on the scalp and axilla, and 5 g/m^2^/h on the trunk and extremities (27). On the thin skin of the female vulva TEWL was measured at 25 g/m^2^/h (27).

The vulvar epithelium exhibits marked regional differences in structure (29). The cutaneous epithelium of the mons pubis and labia majora exhibit a keratinized, stratified, squamous structure similar to skin at other sites. However, skin in this area is more hydrated than skin at other body sites and, therefore, more permeable to some materials and more susceptible to friction effects (30). Moving toward the labia minora, the degree of keratinization, and thickness of the epidermis decreases. The inner third of the labia minora is non-keratinized mucosal tissue (7). The non-keratinized vulvar skin of the labia minora exhibits increased permeability related to the absence of keratin and a loosely packed, less structured lipid barrier (29, 31). In addition, the thinner, inner epithelium represents a shorter distance for penetration of substances (29). Differences in susceptibility to irritant materials seem to be dependent on the relative permeability of the skin of the vulva to the irritant. In addition, vulvar tissue is highly innervated (32). This would be expected to increase the sensations associated with sensitive skin.

## Prevalence of Perceived Sensitive Genital Skin

Few studies have probed perceived sensitivity at multiple anatomic sites among the same group of individuals. Saint-Martory et al. reported on a survey questionnaire study conducted in 2004–2005 among 400 individuals in France (1). The face was most often reported as the site of sensitivity (85% of responders). However, other anatomic sites were also reported as sensitive: the hands (58%), scalp (36%), feet (34%), neck (27%), torso (23%), and back (21%), in order of frequency. The prevalence of some degree of perceived sensitive skin of the scalp has been reported as 24% in the UK, (14) and 32–70% in France (33, 34).

In a study conducted in 2006 in the metropolitan Cincinnati, Ohio area, 1,039 men and women completed a questionnaire related to their perceptions of sensitive skin (16). Within this group, 77% reported some degree of perceived sensitivity of the face, compared with 61% for the body, and 56% for genital skin (16).

### Differences Based on Gender and Ethnicity

**Figure 2:**
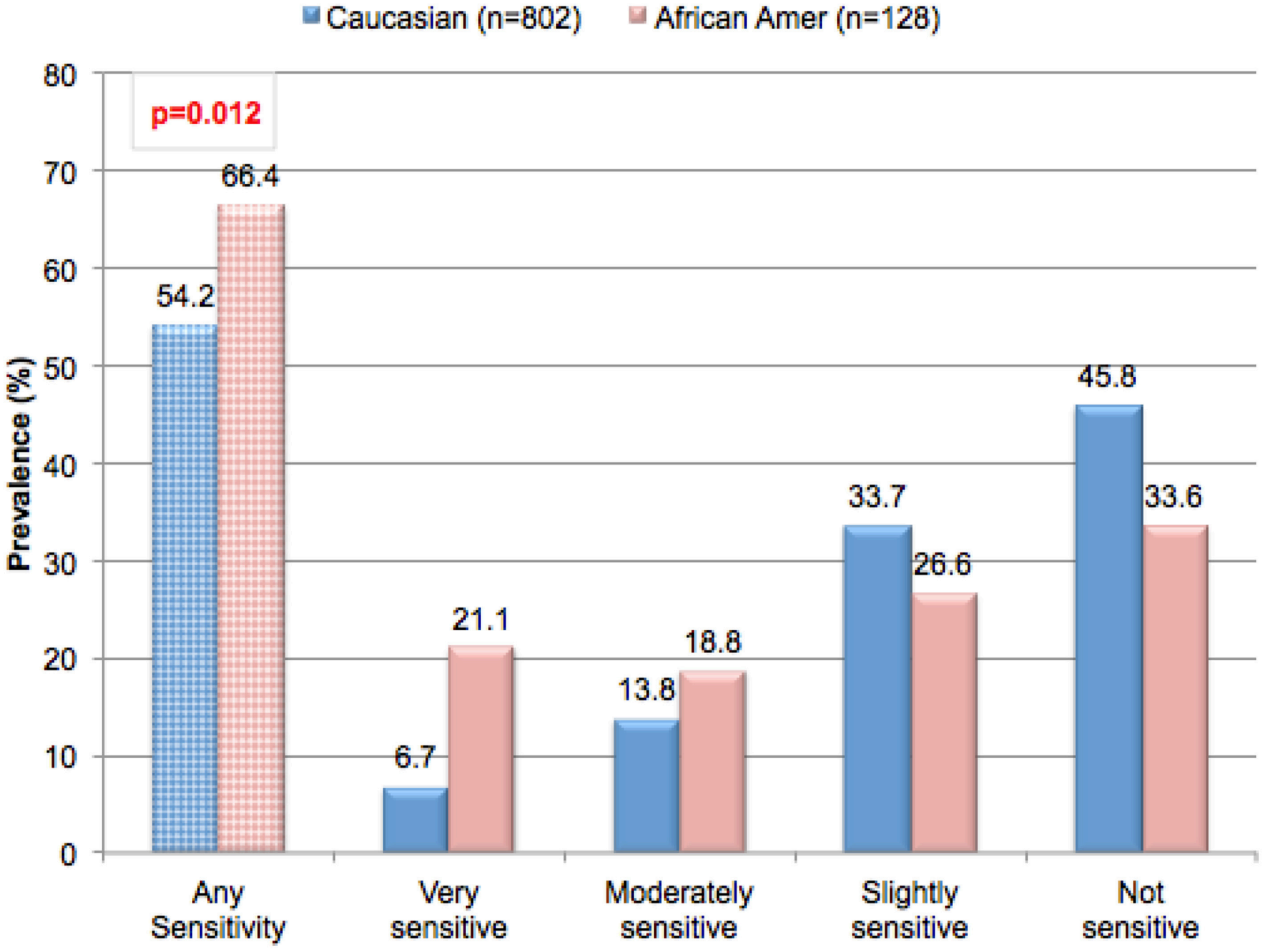
Prevalence of perceived sensitive genital skin among ethnic groups. Subjects in the metropolitan area of Cincinnati, Ohio were asked to complete a sensitive skin questionnaire. Participants were asked to describe their skin sensitivity as very, moderately, slightly or not sensitive. Subsequently, participants were asked to describe the skin of specific anatomic sites including the genital area using the same four-point scale. The percentage of participants claiming any degree of skin sensitivity, and the percentage giving each degree of response (i.e., very, moderately, or slightly) are plotted. Results for ethic groups were compared using a Mantel-Haenszel Chi-Square analysis. [Data adapted from Farage (16)].

Ethnicity appears to play a role in the perception of sensitive genital skin. A significant relationship was found between ethnicity and a perception of sensitive skin in the genital area (Figure 2, *p* = 0.012). In contrast, in the same study, no significant relationships were found between ethnicity and sensitive skin in general, or sensitive skin of the face or body (*p* = 0.15, *p* = 0.24, *p* = 0.13, respectively, data not shown) (16). This is consistent with the findings of Misery et al. in a study conducted in the US (18). These investigators noted that the prevalence of sensitive skin in general was similar among ethnic groups varying slightly from 43% for Caucasians to 52% for African-Americans, with no statistically significant difference (*p* = 0.35). Jourdain et al. conducted a study of perceived sensitive facial skin among a population in San Francisco specifically selected to include approximately equal numbers of 4 ethnicities (15). These authors found no differences between the proportions of women in the 4 ethnic groups who perceived they had some degree of sensitive facial skin (Afro-Americans, 52%; Asians, 51%; Euro-Americans, 50%; and Hispanics, 54%).

Gender differences are also found in the perception of sensitive genital skin. In the study presented in Table 1 (metropolitan Cincinnati, Ohio area), a significantly higher proportion of women (all ethnicities combined) perceived some degree of sensitive genital skin compared to men (58.1% compared to 44.2%, *p* = 0.0009) (16). The gender difference seemed to be driven by the Caucasian subjects who composed the higher proportion of the test population. Among Caucasian subjects 57.0% of women perceived some degree of sensitive genital skin compared to 37.3% of men (*p* < 0.0001) (35). In contrast, among African-Americans there was no difference between genders regarding perceived sensitive genital skin (66.7% of women and 65.0% of men, *p* = 0.84) (35). There is no obvious explanation for why African-American men apparently perceive their genital skin as more sensitive compared to Caucasian men (35).

**Table 1:** Perceptions of self-declared sensitive genital skin by gender and ethnicity.

	**Women**		**Men**		**Comparison of women vs. men**
	**Number**	**Percent**	**Number**	**Percent**	***p*-value**
**All ethnicities (*N* = 1, 032)**	**869**		**163**		
Very sensitive	82	9.4%	6	3.7%	
Moderately	126	14.5%	13	8.0%	
Slightly sensitive	297	34.2%	53	32.5%	
**Sensitive (any degree)**	**505**	**58.1%**	**72**	**44.2%**	**0.0009**
**Not sensitive**	**364**	**41.9%**	**91**	**55.8%**	
**Caucasians (*****N*** **=** **802)**	**684**		**118**		
Very sensitive	51	7.5%	3	2.5%	
Moderately…	101	14.8%	9	7.6%	
Slightly sensitive	238	34.8%	32	27.1%	
**Sensitive (any degree)**	**390**	**57.0%**	**44**	**37.3%**	** < 0.0001**
**Not sensitive**	**294**	**43.0%**	**74**	**62.7%**	
**Afro-Americans (*****N*** **=** **128)**	**108**		**20**		
Very sensitive	26	24.1%	1	5.0%	
Moderately…	20	18.5%	4	20.0%	
Slightly sensitive	26	24.1%	8	40.0%	
**Sensitive (any degree)**	**72**	**66.7%**	**13**	**65.0%**	**0.84**
**Not sensitive**	**36**	**33.3%**	**7**	**35.0%**	

*Statistical comparisons were conducted for sensitivity (any degree) vs. not sensitive (shown in bold type) using a Mantel-Haenszel chi-square test. Other ethnicities (not shown) were not compared statistically due to the low number of participants. [Data adapted from Farage (35, 36)]*.

The overall prevalence of perceived sensitive skin among women was evaluated in three separate studies using the same survey instrument. A first study was conducted in a metropolitan area of the central US (Cincinnati, Ohio), and included 869 women with a mean age of 35.0 years (16, 35–37). Subjects were asked to complete a written questionnaire probing perceptions of sensitive skin. Study participants were not selected based on any criteria related to sensitive skin or hyper-reactivity to consumer products but were participating in unrelated consumer product studies. A second study was conducted in Mississippi using the same written questionnaire (19). In this study, participants were recruited from local organizations with no selection based on any dermatologic or other criteria and were from a predominantly rural environment. The study population consisted of 89 women with a mean age of 45.5 years. In these two studies, the proportions of African-American and Caucasian subjects were similar. A study using a similar protocol and a translation of the same written questionnaire was conducted in China and included 408 women with a mean age of 39 years (24).

**Figure 3:**
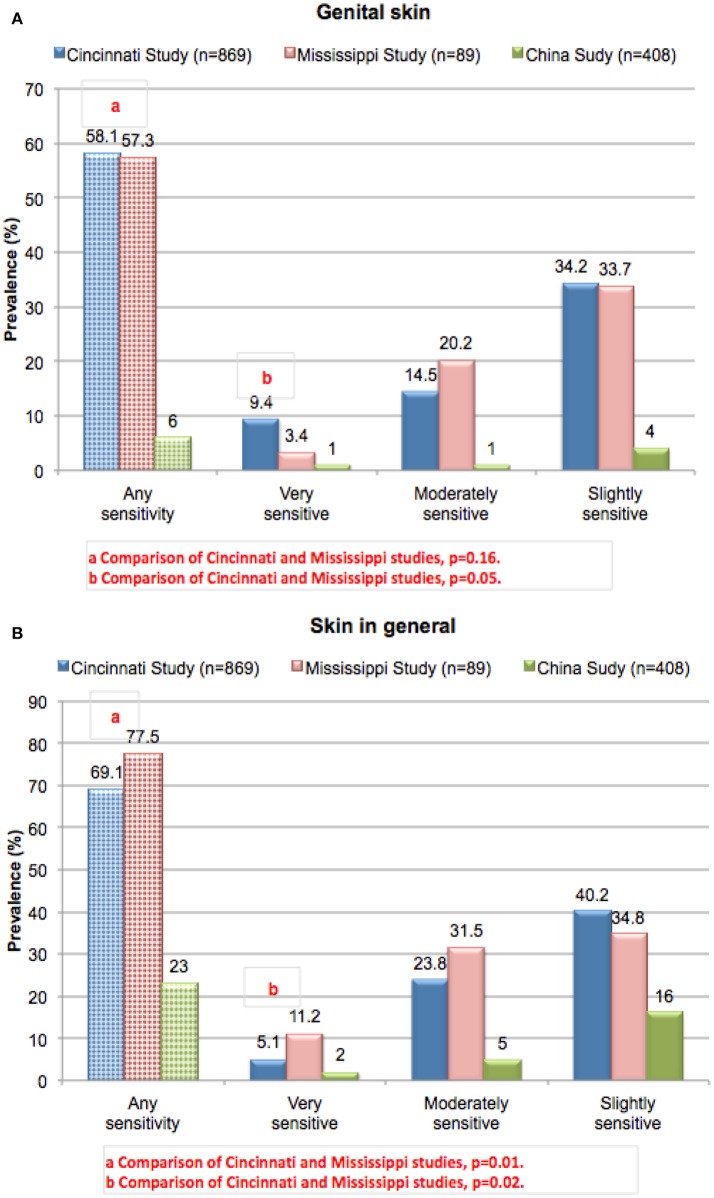
Prevalence of perceived sensitive skin among women. Subjects in three different geographies (urban Ohio, rural Mississippi, and China) were asked to complete the sensitive skin questionnaire described for Figure 2. Results from the Cincinnati and Mississippi studies were compared using a Chi-square analysis. **(A)** Sensitive genital skin, **(B)** sensitive skin in general. [Data adapted from Farage (16) and Farage et al. (19, 24)].

Results from these three studies on women subjects on perceptions of sensitive skin in the genital area and sensitive skin in general are presented in Figures 3A,B, respectively. When the results from the Cincinnati and Mississippi studies were compared, the proportion of subjects who claimed some degree of genital skin sensitivity was not significantly different between the two studies (58.1 and 57.3%, respectively, *p* = 0.16) (19). A higher proportion of subjects from the Cincinnati study claimed their genital skin was very sensitive (9.4 and 3.4%, respectively, *p* = 0.05). When asked about sensitive skin in general (Figure 3B), a slightly higher proportion of subjects in the Mississippi study claimed some degree of sensitivity (77.5% compared to 69.1% from the Cincinnati study, *p* = 0.01) or that the genital skin was very sensitive (11.2% compared to 5.1% from the Cincinnati study, *p* = 0.02). The prevalence of perceived sensitive skin at the specific anatomic sites of the face and body were also slightly higher for the Mississippi study (data not shown). We have reported previously that the proportion of subjects who perceive their genital skin as sensitive increases with age (38). The Mississippi population was older than that surveyed in Ohio (mean ages of 45.5 and 35 years, respectively), so the slight but significant difference in perception of sensitive genital skin cannot be explained by an age difference.

In the study conducted in China (24), the proportion of women who claimed sensitive genital skin was much lower than in both studies in the US at only 6% (Figure 3A). For sensitive skin in general, the prevalence was 23% (Figure 3B). These results are consistent with the observations Xu et al. who reported a lower prevalence of perceived sensitive skin in China compared to Europe and the US (23).

In two other studies conducted in Asia the prevalence of sensitive skin was found to be more consistent with western cultures. Kamide et al. conducted a survey of the general population in Japan. Among the 1,500 of responders, “very” or “rather” sensitive skin was claimed by 54.5% of the entire group (56% of the women and 52.8% of the men) (20). In a study conducted in Korea by Kim et al. among a nationwide sample of 1,000 Koreans, “sensitive” of “very sensitive” facial skin was claimed by 56.8% of the responders (59.2% of women and 54.4% of men) (21).

The Cincinnati and Mississippi studies provide an opportunity to compare different geographic locations within the same country, and to compare a metropolitan vs. rural environment. Neither of these factors appeared to be related to the prevalence of the perception of sensitive genital skin. The two geographic regions have differing climates. Mississippi experiences mild winters, but long summers characterized by high temperatures and high levels of humidity. Cincinnati, Ohio, has mild summers but cold winters. However, when asked about perceived environmental conditions that trigger skin reactions, there were no differences between the two regions with regards to those conditions relevant to sensitive genital skin.

In our survey studies, the questionnaire included lists of external factors (environmental and physiologic conditions) and certain consumer products and asked the responders to indicate if these items ever triggered a skin reaction. In the Cincinnati study (Table 2), a large proportion of the entire study population perceived each of the triggering factors as causing skin reactions on some occasions. However, for the group of individuals claiming some degree of sensitive genital skin the proportion was consistently higher compared those individuals who claimed their skin was not sensitive. Most of the environmental and physiologic conditions (Table 2A) and personal care items (Table 2B) were considered triggering factors by over 50% of the sensitive group, and less than half of the non-sensitive group (36). Dry and cold weather were identified by the majority of both the sensitive and non-sensitive individuals. For the women in the test group, certain feminine products were considered factors that sometimes trigger skin reactions (Table 2C). Comparison of the sensitive to non-sensitive groups indicated that the differences were consistently significant across all potential triggering factors.

**Table 2:** Perceptions about factors triggering skin responses among individuals claiming sensitive genital skin.

	**Sensitive genital skin**		**Genital skin not sensitive**		**Difference between groups**	**Comparison of groups**	
**Potential triggering factors**	**Total responders**	**% Sensitive to factor**	**Total responders**	**% Sensitive to factor**		***p*-value**	**Spearman coefficient**
**(A) ENVIRONMENTAL AND PHYSIOLOGIC CONDITIONS (AMONG MEN AND WOMEN)**							
Rough fabrics	551	**74%**	408	**46%**	28%	< 0.00001	0.32
Hot weather	546	**64%**	408	**43%**	22%	< 0.00001	0.22
Menstrual cycle (women only)	441	**61%**	305	**40%**	21%	< 0.00001	0.22
Stress	548	**61%**	406	**38%**	23%	< 0.00001	0.25
Humid weather	530	**45%**	406	**26%**	19%	< 0.00001	0.20
Dry weather	548	**76%**	413	**63%**	13%	< 0.00001	0.19
Cold weather	553	**84%**	425	**78%**	6%	0.0059	0.099
**(B) PERSONAL CARE ITEMS (AMONG MEN AND WOMEN)**							
Soaps (bar or liquid)	549	**57%**	413	**11%**	46%	< 0.00001	0.47
Undergarments/clothing	551	**68%**	421	**23%**	45%	< 0.00001	0.46
Perfumes/colognes	351	**50%**	406	**36%**	14%	< 0.00001	0.39
Deodorants/antiperspirants	391	**51%**	345	**21%**	30%	< 0.00001	0.31
Toilet paper	533	**31%**	409	**7%**	24%	< 0.00001	0.30
**(C) FEMININE PRODUCTS (AMONG WOMEN ONLY)**							
Menstrual pads	451	**59%**	315	**12%**	46%	< 0.00001	0.45
Feminine wipes	282	**43%**	225	**7%**	36%	< 0.00001	0.41
Douching products	194	**35%**	179	**3%**	32%	< 0.00001	0.40
Panty liners	455	**45%**	325	**8%**	37%	< 0.00001	0.39
Tampons	388	**38%**	296	**4%**	34%	< 0.00001	0.39

*In the Cincinnati study responders were given lists of environmental factors and products and asked to indicate if these items ever caused skin irritation (never, sometimes, frequently, or always). Statistics compared responses of the group claiming some degree of genital sensitivity to those whose genital skin was not sensitive (bold type). Statistical analysis was via a Mantel-Haenszel chi-square test using all levels of perceived irritation frequency (never, sometimes, frequently, or always). These were grouped for presentation in the above table. ND; Not done. [Data on feminine products adapted from Farage (36)]*.

### Effects of Aging

As skin ages, certain physiological changes occur including: reduced epidermal and dermal thickness, reduced hydration, increased permeability, and slower wound healing (39–41). Such changes would lead to the conclusion that skin becomes more susceptible to irritation with aging. However, clinical assessments of responses to irritants indicate that older people tend to be less susceptibility to skin irritation compared to younger individuals (42–46).

In contrast to any changes in the physiological response to irritants with age, the perceptions of general skin sensitivity in western countries do not appear to change with aging. In a phone survey conducted in the US among a nationally representative sample of 994 subjects 45% declared having “sensitive” or “very sensitive” skin (18). There were no significant differences in the prevalence when the data were considered based on age subgroups of 18–24 years, 25–34 years, 35–44 years, 45–54 years, 55–64 years, and ≥65 years. In a survey conducted in the Midwest in a major metropolitan area of the US (Cincinnati, OH), among the 1, 039 subjects, 68% claimed some degree of overall skin sensitivity (16). When subgroups of the responding population were considered, the proportion claiming sensitive skin was 67% for those 30 years and under, 69% for those 31–39 years, 61% for those 40–49 years, and 74% for those 50 years and older. There was no correlation between age and the perception of sensitive skin (*p* = 0.65).

As mentioned earlier, the survey conducted in China indicated there was a statistically significant inverse relationship (*p* < 0.001) between age and the prevalence of reported sensitive or very sensitive skin: 16% in the youngest group (< 25 years), 14% in the middle group (25–49 years), and 10% in the oldest group (≥50 years) (23). Younger age groups may be more aware of the concept of sensitive skin, partially due to beauty-related advertising.

**Figure 4:**
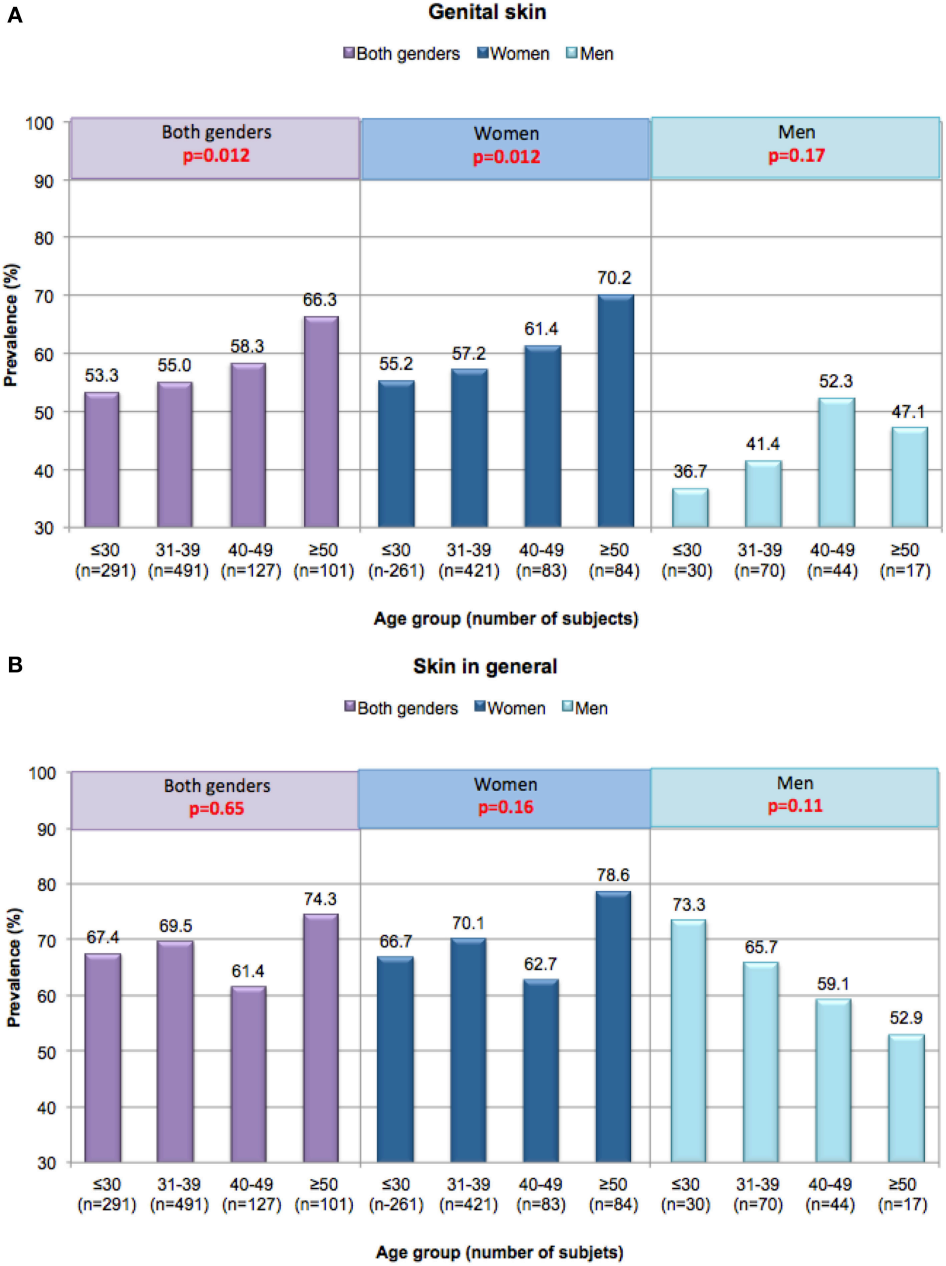
Perceptions of sensitive skin among women and men in different age groups. Responses in the Cincinnati, Ohio study were evaluated based on age group. A Mantel-Haenszel chi-square analysis was conducted to determine if increasing age was associated with an increase in the prevalence of sensitive skin (any degree). **(A)** Sensitive genital skin, **(B)** sensitive skin in general. [Data adapted from Farage (16, 35)].

In the Cincinnati study, the prevalence of sensitive skin of the genital area differed significantly based on age, increasing from 53.3% in the < 30 age group to 66.3% in the >50 age group (*p* = 0.012) (Figure 4A) (16, 36). Among women, sensitive skin of the genital area was more likely to be declared by women age 50 and older (i.e., 70.2% of the age group) than by women in the other age groups (55.2% among women ≤ 30, 57.2% among women 31–39, and 61.4% among women 40–49). The association between age and prevalence was significant among women (*p* = 0.012). Among men there was no apparent association between age and perceived sensitive genital skin (*p* = 0.17) (35). In contrast to the perception of sensitive skin of the genital area, sensitive skin in general does not appear to change with age for either gender (Figure 4B) (16, 36). The differing perceptions among age groups with regards to skin sensitivity of the genital area may be related to specific changes that may occur as a woman ages, such as the onset of menopause.

Table 3 presents the perceptions of the age groups regarding feminine products and the association with skin responses. For all feminine products, individuals with sensitive genital skin in the ≤ 30, 31–39, and 40–49 age groups are more likely to experience skin effects compared to those who do not have sensitive skin (all *p* < 0.005). For the women in the ≥50 age group, all feminine products except tampons are identified as triggering skin responses in a significantly higher proportion of the subjects claiming sensitive genital skin (*p* ≤ 0.02). The small number of responses regarding tampons likely reflects a much smaller proportion of women who use this product in this age group where a substantial portion of the women would be expected to be post-menopausal.

**Table 3:** Perceptions about feminine products perceived to trigger skin responses among women of different age groups.

	**All women in age subgroup**		**Sensitive genital skin**		**Genital skin not sensitive**		**Difference between sensitive and non-sensitive**	**Comparison of sensitive and non-sensitive**	
	**Total responders**	**% Sensitive to factor**	**Total responders**	**% Sensitive to factor**	**Total responses**	**% Sensitive to factor**		***p*-value**	**Spearman coefficient**
**≤30**									
Menstrual pads	246	36%	137	**54%**	109	**13%**	41.2%	< 0.00001	0.42
Panty liners	240	25%	133	**39%**	107	**7%**	32.6%	< 0.00001	0.42
Tampons	229	24%	127	**42%**	102	**3%**	38.8%	< 0.00001	0.42
Feminine wipes	172	22%	90	**39%**	82	**4%**	35.2%	< 0.00001	0.42
Douching products	120	17%	59	**32%**	61	**2%**	30.6%	0.00003	0.41
**31–39**									
Menstrual pads	394	39%	228	**58%**	166	**13%**	45.6%	< 0.00001	0.45
Panty liners	390	31%	223	**47%**	167	**9%**	37.6%	< 0.00001	0.40
Tampons	349	21%	194	**35%**	155	**5%**	29.3%	< 0.00001	0.35
Feminine wipes	220	27%	118	**44%**	102	**8%**	36.3%	< 0.00001	0.40
Douching products	158	20%	74	**37%**	84	**5%**	31.7%	< 0.00001	0.41
**40–49**									
Menstrual pads	72	42%	43	**65%**	29	**7%**	58.2%	0.00001	0.56
Panty liners	76	37%	46	**52%**	30	**13%**	38.9%	0.0005	0.41
Tampons	64	31%	39	**49%**	25	**4%**	44.7%	0.001	0.44
Feminine wipes	54	24%	30	**33%**	24	**13%**	20.8%	0.001	0.44
Douching products	43	14%	26	**23%**	17	**0%**	23.1%	0.005	0.29
**≥50**									
Menstrual pads	46	54%	34	**68%**	12	**17%**	50.9%	0.001	0.45
Panty liners	59	37%	44	**48%**	15	**7%**	41.0%	0.001	0.45
Tampons	27	22%	19	**32%**	8	**0%**	31.6%	0.12	0.34
Feminine wipes	53	47%	41	**56%**	12	**17%**	39.4%	0.02	0.34
Douching products	43	30%	31	**42%**	12	**0%**	41.9%	0.02	0.40

*Responders were given lists of personal items and products and asked to indicate if these items ever caused skin irritation. A Mantel-Haenszel chi-square test was used to compare Statistics compared responses of the group claiming some degree of genital sensitivity to those whose genital skin was not sensitive (bold type)*.

Findings regarding perceived sensitive skin and aging have been mixed. For example, in a study conducted in the US, Misery et al. concluded that overall sensitivity does not vary with age (18). However, these investigators also reported that perceived sensitivity of the scalp increased with age (34). In a study in France, (47) reported that the prevalence of perceived sensitive skin of the face decreased with age for both women (67% in 35–39 year-old, to 55% in 55–60 year-old) and men (35% in 45–49 year-old, to 29% in 55–60 year-old). In a study conducted in Mexico, Hernández-Blanco et al. did not see a trend with regards to the incidence of self-diagnosed sensitive skin and age (22). We have found that sensitive skin of the face and body does not appear to change with age (38).

Taken as a whole, these results serve as a reminder that sensitive skin continues to be a complex problem involving a complicated interplay of physiological, psychological and cultural factors. As we unravel this phenomenon the importance of understanding differences between ethnicities, gender, age group and anatomic site is becoming increasingly clear.

### Effects of Incontinence

Urinary incontinence is extremely common among women. Reports vary with regards to the precise percentage of the female population who suffer from incontinence. In a study conducted in Sweden among 3,071 women, Hagglund et al. reported an overall prevalence of 26%, with a prevalence of 12% among women under 30 years of age (48). In 1988 Jolleys reported an overall prevalence of urinary incontinence of 41% in a survey among 833 women in the UK, (49) whereas Thomas et al. reported that 16.6% of women reported occasional incontinence, (50) and 8.5% reported regular incontinence in a survey of 9,323 women in the London area.

The risk of incontinence increases with age (51, 52). In a review of relevant literature, Botlero et al. reported that the prevalence among younger women who had at least one episode of urinary incontinence within the previous year was about 13%, compared to 46% among women in their 50's and 60's (53). Brown et al. reported a prevalence of 28% among a cohort of 2,763 participants in survey of post-menopausal women (54). Roberts et al. reported on the results of a community-based study involving 762 women and 778 men (55). The mean age (±SD) of the subjects was 65.9 (±9.2) and 66.3 (±9.2), respectively. The prevalence of urinary incontinence was 48.4% among women and 25.6% among men. In a study conducted in the UK among 314 randomly selected female patients at a health promotion clinic, the prevalence of incontinence was 53.2% for the entire test population, with an incidence of 34.7% among women 20–29 years of age, and over 50% in women in age groups spanning 40–79 years of age (56). In contrast, 21,590 male heads of household in the US participated in a survey to determine whether the respondent had symptoms of urinary incontinence (57). Overall, 12.7% reported symptoms during the previous 30 days. The association between urinary incontinence and age was significant, with prevalence among the youngest age group (men 18–34 years) at 7.2%, and among the oldest age group (men ≥75 years) at 30.2%.

**Figure 5:**
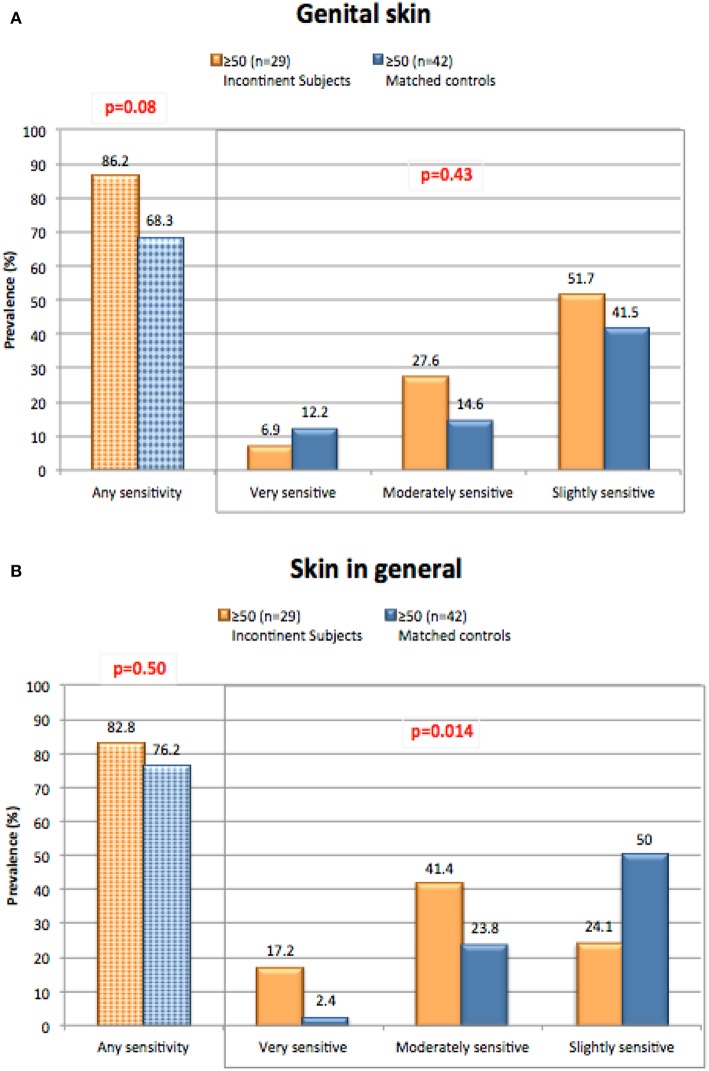
Perceptions of sensitive skin among women with incontinence. The sensitive skin questionnaire was administered to women who suffered from light urinary incontinence age 50 and above. Responses were compared to age matched control subjects who do not have incontinence. The percentage of participants claiming any degree of skin sensitivity, and the percentage giving each degree of response (i.e., very, moderately, or slightly) are plotted. Results were compared for any degree of sensitivity using a Chi-square analysis, and for all three degrees of sensitivity using a Mantel-Haenszel chi-square analysis. **(A)** Sensitive genital skin, **(B)** sensitive skin in general. [Data adapted from Farage (17)].

We conducted a study to evaluate perceptions of sensitive skin in women with urinary incontinence compared to a group of age-matched controls (17). The participants included women who suffered from light urinary incontinence age 50 and above who participated in focus groups as part of development efforts aimed toward incontinence products. Responses were compared to age matched control subjects who do not have incontinence. Results are presented in Figure 5. We expected the incontinent subjects might have an increased perception of sensitive skin in the genital area since these individuals may experience periodic wetness and may wear pads or incontinence products to control wetness. However, such was not the case. A directionally higher proportion of incontinent women reported some degree of sensitive genital skin (very, moderately or slightly) compared to the controls (86.2 and 68.3%, respectively), but the difference between this group and the age-matched control group was not significant (*p* = 0.08) (Figure 5A). A directionally lower percentage of incontinent subjects described their genital skin as “very” sensitive compared to the control subjects (6.9 and 12.2%, respectively, *p* = 0.08). It is possible that incontinent individuals may attribute adverse sensory effects or irritation to their incontinent status, rather than to the notion that they have sensitive skin of the genital area (36). With regards to sensitive skin in general (Figure 5B), there was no difference between the test populations in the proportion claiming some degree of sensitivity (82.8% or incontinent subjects, and 76.2% of control subjects, *p* = 0.50). Interestingly, the incontinent women were directionally more inclined to describe their skin in general as “very” or “moderately” sensitive (*p* = 0.014). There are no apparent reasons for this tendency.

A large proportion of both study groups perceived each external factor as causing skin responses on some occasions (Table 4). With regards to environmental and physiologic factors, there were no significant differences between groups except for “cold weather” (Table 4A). Note that “menstrual cycle” was not applicable to most of the individuals in this study since the study population was age 50 and above, therefore, there were few responses reported for this factor. For the personal care items and feminine products (Tables 4B,C, respectively), there were no differences between incontinent and control groups (17).

**Table 4:** Perceptions about skin responses due to relevant external factors among women with incontinence.

	**Incontinent (*****n*** **=** **29)**		**Control (*****n*** **=** **42)**		***p*-value**
	**Total responses**	**% Sensitive to factor**	**Total responses**	**% Sensitive to factor**	
**(A) ENVIRONMENTAL AND PHYSIOLOGIC CONDITIONS**					
Rough fabrics	27	84%	38	81%	0.45
Hot weather	24	87%	34	83%	0.25
Stress	26	58%	36	47%	0.57
Menstrual cycle	8	13%	18	22%	1
Humid weather	27	41%	36	61%	0.38
Dry weather	27	71%	37	72%	0.28
Cold weather	27	100%	34	83%	**0.033**
**(B) PERSONAL CARE ITEMS**					
Soaps (bar or liquid)	28	57%	38	66%	0.72
Undergarments/clothing	28	47%	34	56%	0.81
Perfumes/colognes	22	68%	25	60%	0.71
Deodorants/antiperspirants	21	53%	27	59%	1
Toilet paper	27	37%	33	36%	0.81
**(C) FEMININE PRODUCTS**					
Menstrual pads	18	61%	17	53%	0.60
Feminine wipes	23	39%	20	60%	0.19
Douching products	14	28%	17	47%	0.79
Panty liners	24	38%	22	37%	1
Tampons	8	26%	10	20%	0.72

*Responders were given lists of environmental factors and products and asked to indicate if these items ever caused skin irritation. A Mantel-Haenszel chi-square test was used to compare responses of the group claiming some degree of genital sensitivity to those whose genital skin was not sensitive. The p-value indicating significance is in bold type. [Data adapted from Farage (17)]*.

## Effects of Menopause: Differences in Biomolecular and Physical Measures of the Urogenital Skin

Falcone et al. reported the results of a digital questionnaire distributed to a population of women aged 20–65 years old (58). Among the 278 responders, 121 were premenopausal, 55 were perimenopausal and 102 were post-menopausal. Sensitive skin of the face was reported by 54% of responders, while sensitive skin of the genital area was reported by only 10.8%. Interestingly, the responses for skin in the genital area did not differ significantly for life stage. However, for sensitive facial skin there were significant differences (*p* = 0.02), with 62% of premenopausal women claiming sensitivity compared to 54% of perimenopausal and 43% of post-menopausal women.

We conducted a study to evaluate potential differences in biomolecular and physical measures of the urogenital skin among women in different stages of life (59, 60). Subjects were categorized into three groups of 15 subjects each: the Pre-M group consisted of pre-menopausal women (mean age ± SD = 33.0 ± 6.4); the Post-M Non-HRT group consisted of post-menopausal women who were not receiving any hormone replacement therapy and who exhibited a vaginal atrophy score ≥6 and vaginal pH ≥5 (60.7 ± 3.6); and the Post-M HRT group consisted of post-menopausal women who had been taking some form of hormone replacement therapy for a minimum of 1 year (60.5 ± 3.6). Evaluations were conducted on three genital sites; introitus, labia minora and labia majora. Physical measures at these sites included skin temperature and pH. In addition, sequential tape strips were used to collect material for the quantitative analysis of a variety of biomarkers and cytokines. We also collected information about perceived sensitive skin and urogenital symptoms.

Group sizes in this study were relatively small, resulting in a low likelihood of significant differences between groups or observations. However, some interesting trends emerged that provide directions for further investigation regarding an understanding of sensitive skin.

**Figure 6:**
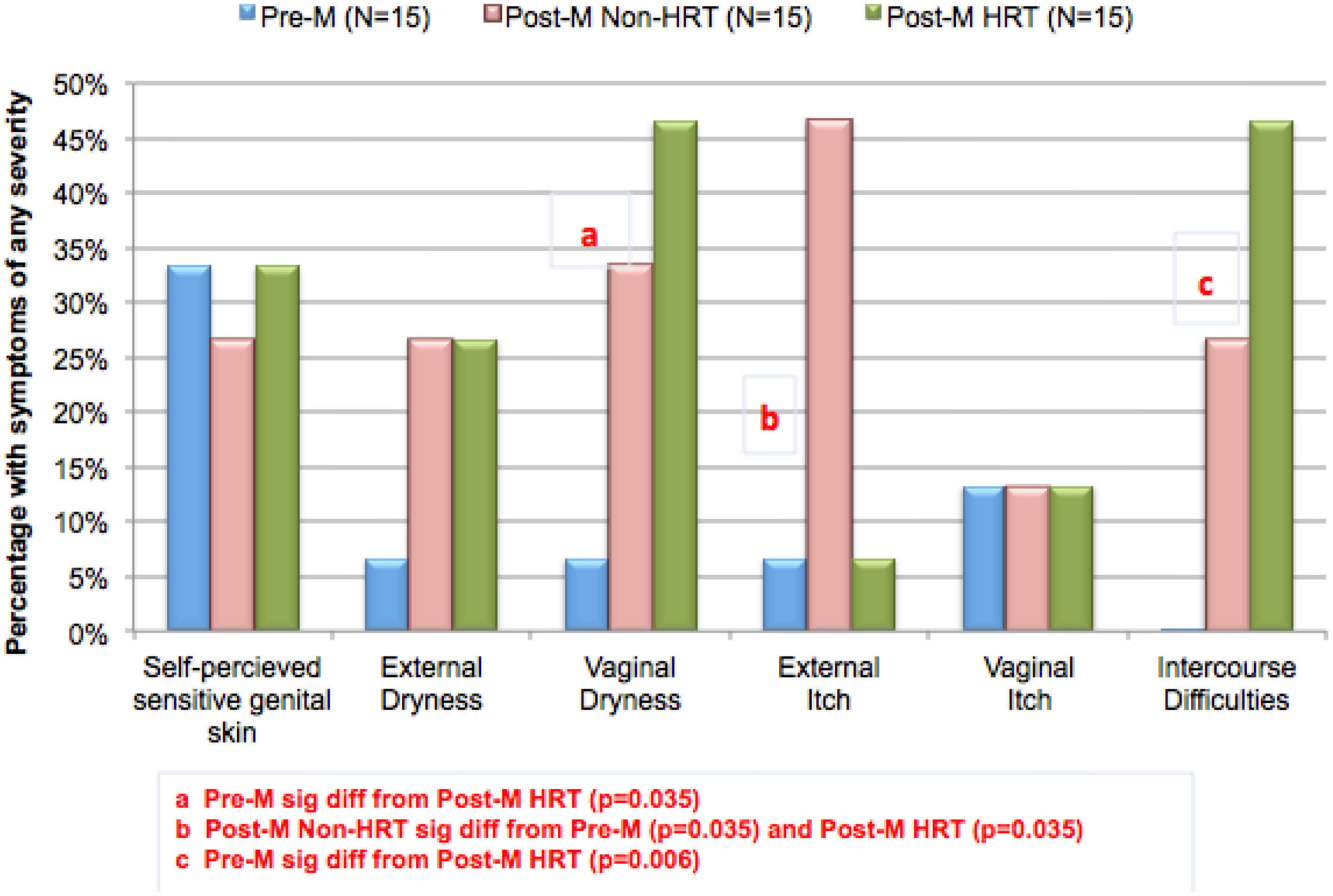
Changing perceptions after menopause. A study was conducted to evaluate potential differences in biomolecular and physical measures of the urogenital skin among women in different stages of life. Participants were asked about perceptions of sensitive genital skin, and about specific subjective symptoms (i.e., external and vaginal dryness, external and vaginal itch, or difficulties with intercourse). The groups (15 each) consisted of women who were: pre-menopausal (Pre-M), post-menopausal on no hormone replacement therapy (Post-M Non-HRT), and post-menopausal receiving hormone replacement therapy (Post-M HRT). The proportion of individuals in each test group claiming any degree of sensitive genital skin or any of the subjective symptoms is plotted. Pairwise comparisons were conducted using Fisher's exact test.

Figure 6 shows results of the perception of sensitive genital skin, and the presence of subjective symptoms in the three test groups. The proportion of this population who perceived they had sensitive skin of the genital area was smaller in this study compared to previous studies in the same geographic area (Cincinnati, Ohio), i.e., about one third in this study compared to 58% (shown in Table 1). There is no apparent reason for this difference; however, it may be related to the small sample size (15 individuals per group). As expected, symptoms associated with vulvovaginal atrophy (i.e., dryness, itch and difficulties with intercourse) were reported by very few of the Pre-M subjects. Compared to the Pre-M group, a directionally higher proportion of both Post-M groups (HRT and Non-HRT) reported external and vaginal dryness, and difficulties with intercourse. The differences between the Pre-M group and the Post-M HRT group were significant for vaginal dryness (*p* = 0.035) and intercourse difficulties (*p* = 0.006). External itch was reported by a significantly higher proportion of the Post-M Non-HRT group compared to both the Pre-M and Post-M HRT groups (*p* = 0.035 for each comparison).

**Figure 7:**
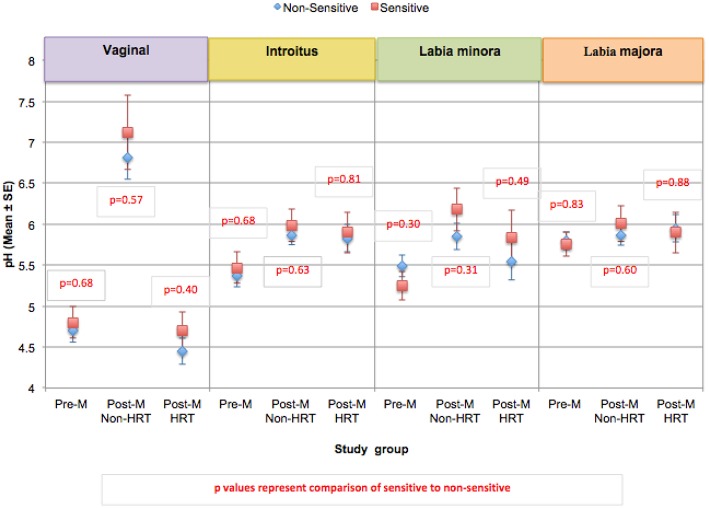
Comparison of skin pH at genital sites among women with sensitive genital skin. In the study to evaluate biomolecular and physical measures of the urogenital skin, evaluations of skin pH were conducted at the following anatomic sites; vaginally, at the introitus, the labia minora, and the labia majora. For each group, the mean pH for those who did not have sensitive genital skin was compared to those declaring sensitive skin. The number declaring sensitive genital within each group was: Pre-M, 5; Post-M Non-HRT, 4; Post-M HRT, 5. A mixed linear model was used to analyze the pH at different anatomic sites. None of the comparisons were significantly different (i.e., *p* ≤ 0.05).

The pH was evaluated at 4 anatomic sites; vaginally and at the introitus, the labia minora, and the labia majora (60). Differences in pH were small (not reaching statistical significance), however within each test group, women who claimed sensitive genital skin tended to demonstrate a higher pH vaginally and at the introitus compared to those who did not claim sensitive skin (Figure 7). As expected, HRT appeared to result in a vaginal pH that was close to that of the Pre-M group. As mentioned, differences among this small test population did not reach significance, but an interesting area for future research with a larger number of subjects would be an investigation into whether pH is related to sensitive genital skin, and the impact of HRT on the pH of genital tissues other than the vagina.

**Figure 8:**
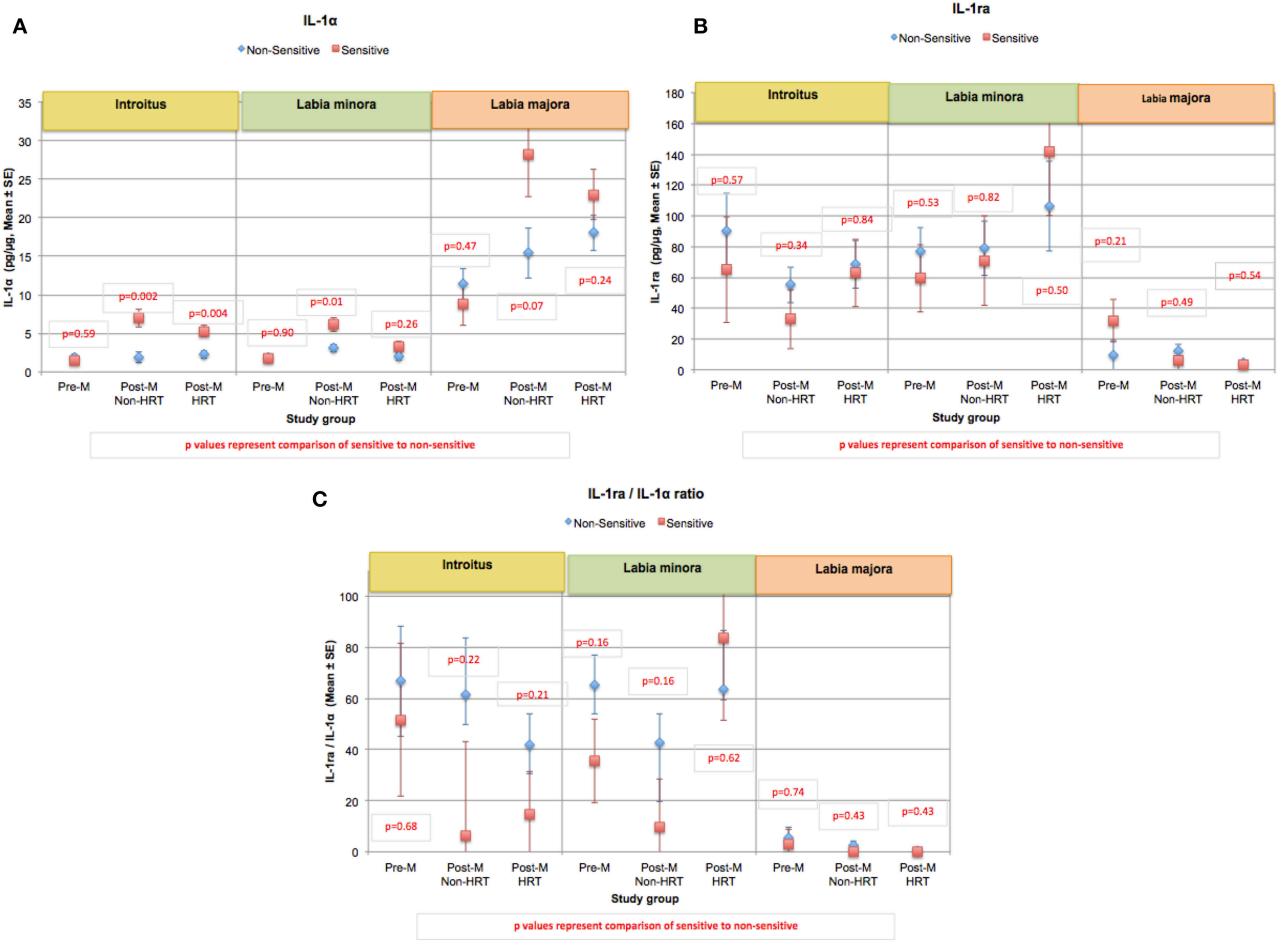
Content of IL-1α, IL-1ra, and the ratio of IL-1ra/IL-1α at genital sites among women with sensitive genital skin. In the study to evaluate biomolecular and physical measures of the urogenital skin, sequential tape strips were used to collect material for the quantitative analysis of a variety of biomarkers and cytokines. Results of analyses of: **(A)** IL-1α, **(B)** IL-1ra, and **(C)** the ratio of IL-1ra/IL-1α are presented for each group. For each group, the mean value for those who did not have sensitive genital skin was compared to those declaring sensitive skin using a mixed linear model. **(A)** Mean IL-1α, **(B)** mean IL-1ra, **(C)** mean of the ratios of IL-1ra/IL-1α.

The content of IL-1α, IL-1ra, and the ratio of IL-1ra/IL-1α were evaluated in the study groups (Figure 8) (60). In both post-menopausal study groups, the IL-1α content recovered from tape stripping at the introitus was significantly higher in women claiming sensitive genital skin (Post-M Non-HRT, *p* = 0.002; Post-M HRT, *p* = 0.004) (Figure 8A). For those claiming sensitive skin in the Post-M Non-HRT group, the IL-1α content was also significantly higher at the labia minora (*p* = 0.01). There were no significant differences in the IL-1ra content or the ratio of IL-1ra/IL-1α when individuals with sensitive genital skin were compared to those without (Figures 8B,C, respectively).

The cytokine IL-1α is produced by epithelial cells, and the normal human epidermis acts as a major reservoir of this material. Regulated cytokine expression is essential to the quality and function of the epidermal barrier, and deregulation of this complex signaling mechanism can result in multiple consequences in skin barrier function (61). The cytokine IL-1ra functions as a competitive inhibitor to block the response to IL-1α (62). There is evidence that levels of IL-1α and IL-1ra measured in the stratum corneum may be related to inflammation. Hirao et al. reported that the stratum corneum of an area of skin unexposed to sunlight, i.e., the inner side of the upper arm, contained more IL-1α than a sun exposed area, i.e., the face. In contrast, the IL-1ra content was reversed, with the sun-exposed area containing higher amounts than the unexposed area (63). The ratio of IL-1ra to IL-1α was over 100 in the sun-exposed area, and only 8 in the unexposed area, leading to the conclusion that IL-1ra activity was predominant in sun exposed areas, and IL-1α was predominant in unexposed areas. These same authors reported that the IL-1α content in the unexposed site increased with age, while the content of IL-1ra decreased, resulting in an age-dependent decrease in the IL-1ra/IL-1α ratio (63). In infants suffering from diaper rash, Perkins et al. reported a positive correlation between IL-1ra levels recovered from the buttocks and diaper rash severity (64). The ratio of IL-1ra/IL-1α for sun-exposed skin (i.e., skin on the face and lower leg) was significantly higher (3–6 times, respectively) than skin that was minimally sun-exposed (upper back, underarm, upper leg) (64).

**Figure 9:**
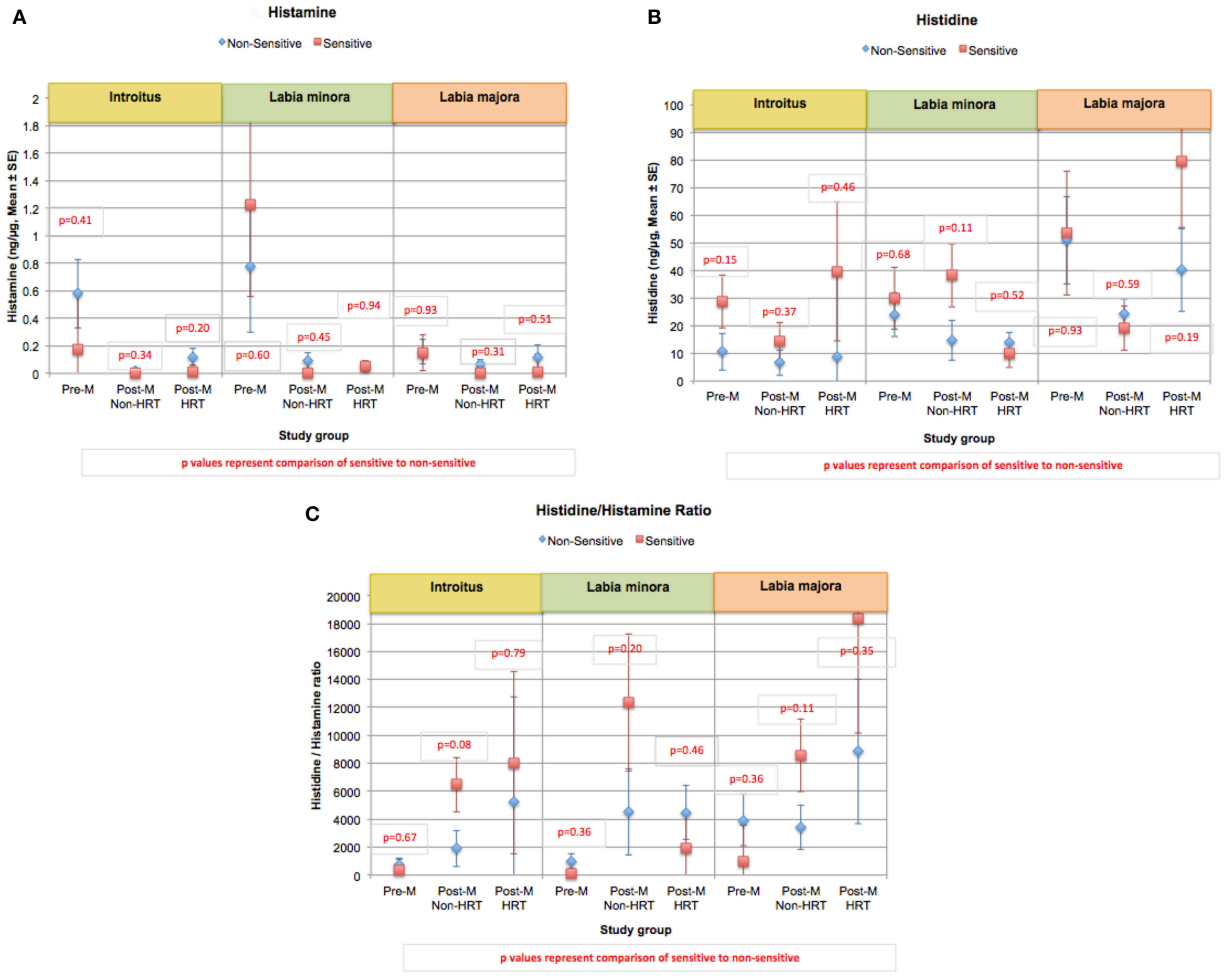
Content of histamine, histidine, and the ratio of histidine/histamine at genital sites among women with sensitive genital skin. In the study to evaluate biomolecular and physical measures of the urogenital skin, histamine, histidine, and the ratio of histidine/histamine were evaluated. Results of analyses of the Pre-M and Post-M Non-HRT groups are presented for those who did not have sensitive genital skin compared to those declaring sensitive skin using a mixed linear model. **(A)** Mean histamine, **(B)** mean histidine, **(C)** mean of the ratios of histamine/histidine.

Histamine is derived from the decarboxylation of the amino acid histidine (65). An altered ratio of histamine to histidine may indicate a change in the induction of histidine decarboxylase or a shift in the equilibrium between these two materials. Overall histamine and histidine levels did differ significantly when individuals with perceived sensitive genital skin were compared to non-sensitive individuals (Figures 9A,B). Previously we reported that the ratio of histidine to histamine was significantly higher at the introitus and labia majora in individuals with perceived sensitive genital skin compared to individuals who were non-sensitive (60). However, after this publication appeared, corrected statistical analyses indicated that, although individuals with sensitive skin tended to have higher ratios of histidine to histamine, the differences were not significant (Figure 9C).

Histamine is commonly associated with itch in a dose-dependent manner. Generally, we did not see an increase in histamine levels among the Post-M Non-HRT group of women who had a higher number of complaints of itching. This provides an indirect indication that itching experienced by these women may be caused by other biochemical mediators associated with itch, and/or other stimuli, such as dryness (66). Several roles have been identified for histamine that are related to sexual function (65). Histamine receptors are important in brain areas involved in sexual arousal (67). As a neurotransmitter, histamine levels are related to sexual desire; a decrease in histamine causes a decrease in sexual desire, and an increase causes the reverse (68). Histamine has local effects on smooth muscle and blood vessels critical to physiological sexual arousal (69). In women, this involves an increase in clitoral cavernosal artery inflow and an increase in clitoral intracavernous pressure that leads to tumescence and extrusion of the clitoris (68). Engorgement of the genital vascular network increases pressure inside the vaginal capillaries and results in lubrication of the epithelial surface of the vaginal wall (68). Histamine also causes the sexual flush that occurs during arousal. Orgasm is triggered when histamine is released from the mast cells in the genitals. For some women who fail to achieve sexual pleasure and orgasm the problem may be a result of a biochemical imbalance related to histamine and histidine. Further, histamine may be an important biomarker for genital tissue health regarding blood perfusion and sexual function.

## Unique Habits and Practices Effecting Genital Skin

There are several aesthetic practices that can impact the skin of the genital area. Pubic hair grooming, mostly consisting of hair removal, is the most common practice. Recent investigations indicate that well-over half the population in the United States engage in the practice. Table 5 summarizes three studies conducted on grooming practices in the United States. Two were conducted using female panelists (70, 71), and one was conducted among women and men (72). All three studies determined that the most popular methods of pubic hair removal among women is a non-electric razor (~60–90% of groomers). In the study that included men as subjects it was determined that both non-electric and electric razors were commonly used for pubic hair removal (30.1 and 35.9%, respectively) (72).

**Table 5:** Pubic hair grooming habits.

	**DeMaria and Berenson (70)**	**Rowen et al. (71)**	**Truesdale et al. (72)**	
	**Adults 16–40**	**Adults 18–65**	**Adults 18–65**	
**Year(s) study conducted**	**2010–2011**	**2012–2013**	**2014**	
Number of subjects	1,677 women	3,316 women	3,372 women	4,198 men
Number of subjects	1,677	3,316	3,372	4,198
Prevalence of grooming	71.7%	83.8%	83.5%	66.5%
**Method**				
Non-electric razor	92.2%	61.0%	70.5%	30.1%
Electric razor	*Not given*	12.0%	14.3%	35.9%
Scissors	27.6%	17.5%	17.4%	15.7%
Waxing	19.5%	4.6%	6.6%	0.4%
Depilatory creams	22.3%	1.2%	*Not given*	*Not given*
Laser or electrolysis	13.9%	0.8%	1.1%	0.3%
Other	*Not given*	1.7%	3.1%	2.0%
Pubic hair dye	2.8%	*Not given*	*Not given*	*Not given*

All studies reported that the occurrence and frequency of pubic hair grooming is higher among younger individuals (70, 71, 73). In a study of university students conducted in 2015 by Butler et al. the prevalence was about 95% of women and 85% of men in the younger group (74).

Rowen et al. reported that the most common sites of hair removal for women were around the vagina (75.1%), above the vagina (73.9%), and inner thighs (54.2%) (71). For women the most common reason given for hair removal was hygienic purposes (over 50%), followed by attractiveness (31%), partner preference (21%), and ease of oral sex (20%) were also given (71). The majority of male groomers remove the hair above the penis (82–91%, depending on age group), followed by the scrotum (57–71%), penile shaft (51–61%), and inner thigh (25–45%) (73). The majority of men report grooming in preparation for sexual activity (50–72%, depending on age group), followed by hygiene (61%), and routine care (44%) (73).

Complications from removing pubic hair are generally minor and include abrasions, itching, cuts, and rash (74, 75). DaMaria et al. reported that a majority (over 90%) of complications related to pubic hair removal occurred among women who had shaved with a razor (75).

Other types of hair removal methods include waxing, depilatory creams, laser hair removal and electrolysis. Several depilatories and home waxing products are formulated specifically for use on the “bikini line” and not on other areas of the genitalia. Use on areas other than the bikini line, such as the vulva, can lead to irritation. Electrolysis and laser hair removal are newer approaches to permanent hair removal and should only be done by professionals.

Some individuals resort to dyeing since pubic hair tends to be darker than hair color and grays with age. Home hair coloring products are not formulated for use on the vulva and would likely cause irritation if used for that purpose. A professional colorist and a dye formulated for facial hair are the best choices.

Other aesthetic modifications include piercings and tattoos. Little is published on complications resulting from these practices. Genital piercings can provide an environment for local microflora to multiply, potentially increasing sexually transmitted infections (76). Male genital piercings have been associated with complications such as urethral rupture or obstruction, paraphimosis, and scar formation. Female genital piercings may damage condoms or dislodge diaphragms during sexual intercourse, thereby leading to a higher risk of pregnancy.

## Conclusion

Sensitive skin is a real phenomenon affecting a large proportion of the population, and it is becoming increasingly clear that individuals can have different perceptions about the sensitivity of their skin based on anatomic site. For women, sensitive skin of the genital area can have an adverse impact on daily life and activities. Aging can contribute to the prevalence and symptoms of sensitive skin due to the normal changes that occur to epidermal structure and function. Added to that, aging results in an increased likelihood of incontinence among women, and the inevitable onset of menopause with several changes.

As the population ages, it will become increasingly important to understand the phenomenon of sensitive skin to develop effective therapies for those who suffer from it. We are only beginning to understand the physiological basis for this condition. Painstaking evaluation of the physical and biochemical properties of sensitive skin is a next step in illuminating the mechanisms and causes of this condition.

## Author Contributions

The author confirms being the sole contributor of this work and has approved it for publication.

### Conflict of Interest Statement

MF is an employee of The Procter and Gamble Company.
